# Effect of Printing Angle on Mechanical Properties of Fused Filament Fabrication (FFF)-Printed Polymers for Below-Knee Prosthetic Sockets: An Experimental and Finite Element Analysis Study

**DOI:** 10.3390/polym18192431

**Published:** 2026-10-06

**Authors:** Ismail Boudjemaa, Omar Khatir, Abdelkader Benkhettou, Bel Abbes Bachir Bouiadjra, Abdrahmene Sahli, Murat Yaylacı, Dursun Murat Sekban, Yılmaz Güvercin, Ecren Uzun Yaylacı, Muhammet Kalkışım

**Affiliations:** 1 Gaseous Fuels and Environment Laboratory, Department of Mechanical Engineering, University of Sciences and Technology of Oran Mohamed Boudiaf (USTO-MB), Oran 31000, Algeria; ismailboubou000@gmail.com; 2 Laboratory Mechanics Physics of Materials (LMPM), Department of Mechanical Engineering, University of Sidi Bel Abbes, Sidi Bel Abbes 22000, Algeria; khatiromar8@gmail.com (O.K.); aekben.dz22@gmail.com (A.B.); bachirbou@yahoo.fr (B.A.B.B.); sahliabderahmen@yahoo.fr (A.S.); 3 Department of Civil Engineering, Recep Tayyip Erdogan University, Rize 53100, Türkiye; 4 Biomedical Engineering MSc Program, Recep Tayyip Erdogan University, Rize 53100, Türkiye; 5 Dijitalpark Teknokent, Murat Yaylacı-Luzeri R&D Engineering Company, Rize 53100, Türkiye; 6 Department of Marine Engineering Operations, Karadeniz Technical University, Trabzon 61080, Türkiye; msekban@ktu.edu.tr; 7 Trabzon Teknokent, WMS Engineering Services Industry Trade Limited Company, Trabzon 61080, Türkiye; 8 Department of Orthopaedic and Traumatology, Trabzon University, Trabzon 61030, Türkiye; yilmaz.guvercin@erdogan.edu.tr; 9 Faculty of Fisheries, Recep Tayyip Erdogan University, Rize 53100, Türkiye; ecren.uzunyaylaci@erdogan.edu.tr; 10 Department of Orthopaedics, School of Medicine, Karadeniz Technical University, Trabzon 61080, Türkiye; muhammetkalkisim@gmail.com

**Keywords:** 3D printing, printing orientation, tensile properties, finite element analysis, prosthetic socket

## Abstract

This study investigates the influence of printing angle on the mechanical performance of 3D-printed prosthetic sockets through an integrated experimental and computational approach. Tensile tests were conducted on Acrylonitrile Butadiene Styrene (ABS), Polylactic Acid (PLA), and enhanced PLA (PLA+) specimens fabricated at four orientations (0°, 30°, 60°, and 90°). The results confirmed clear printing orientation dependence, with Young’s modulus and ultimate tensile strength varying across printing angles in a material-specific manner. The experimentally determined Young’s modulus for each material–orientation combination was implemented as an equivalent isotropic elastic input in a separate finite element simulation of a patient-specific transtibial socket under simplified quasi-static stance loading. The FE simulations revealed that PLA+ printed at 0° provided a balanced structural response: it maintained von Mises stress within the socket at 1.98 MPa, limited displacement to 0.29 mm, and kept interface contact pressure at 0.089 MPa. In contrast, the more compliant ABS, particularly at a 90° orientation, exhibited a higher displacement of approximately 0.37 mm, which may influence socket compliance. This research provides quantitative, data-driven guidelines for the additive manufacturing of load-bearing medical devices, demonstrating that precise control of print orientation and material selection is essential for engineering prosthetic sockets that balance structural support with patient-specific interface requirements.

## 1. Introduction

The evolution of lower-limb prosthetic devices has significantly enhanced the mobility and quality of life for individuals with below-knee amputation (BKA) [1]. The prosthetic socket, as the critical interface between the residual limb and the artificial device, plays a paramount role in this system. Its primary function is to ensure secure load transfer, stability, and comfort during ambulation [2]. An ill-fitting or poorly designed socket can lead to a cascade of clinical complications, including soft tissue damage, pain, dermatological issues, and ultimately, socket rejection and user abandonment of the prosthesis. Consequently, the optimization of socket design and manufacturing is a persistent and central challenge in prosthetic rehabilitation.

The biomechanical performance of the socket is intrinsically linked to the properties of its constituent materials. A material’s stiffness, strength, flexibility, and fatigue resistance directly influence stress distribution across the sensitive residual limb [3]. Appropriate material selection can mitigate the development of interfacial stresses, particularly peak pressures and shear forces, which are primary contributors to skin breakdown and patient discomfort [4]. Historically, socket fabrication has relied on manual, labor-intensive techniques using materials like thermoplastics and laminated composites, which, while customizable, often lead to inconsistencies and prolonged production times [5].

Additive manufacturing (3D printing) has emerged as a transformative technology in prosthetics, promising unprecedented levels of customization, repeatability, and design freedom for creating patient-specific sockets [6,7]. Fused Filament Fabrication (FFF), utilizing polymers such as Acrylonitrile Butadiene Styrene (ABS), Polylactic Acid (PLA), and its enhanced variants (PLA+), is particularly accessible and cost-effective. However, a significant challenge arises from the inherent anisotropy of FFF parts, where mechanical properties are highly dependent on the direction of the deposited layers relative to the applied load [8]. This anisotropy, governed primarily by printing orientation, can lead to different structural responses if it is not controlled. Recent material-extrusion studies further show that flow-field control, print-path design, filler orientation, and polymer blending can strongly influence the resulting mechanical or functional response [9,10,11]. Across engineering applications, experimentally determined material properties are commonly incorporated into numerical models to evaluate how process-induced variations influence contact response, stress redistribution, and component-level structural performance [12,13,14,15,16,17,18].

While existing literature has characterized the bulk mechanical properties of 3D-printed polymers [19], and FEA is widely employed to analyze socket-limb interaction [20], a critical gap persists. There is a lack of integrated studies that systematically quantify the effect of specific printing angles on the fundamental tensile properties of common FFF materials and directly translate these empirical, angle-dependent data into a functional FEA model of a BKA socket. Most FEA studies assume a single isotropic material definition independent of printing orientation, which does not capture the manufacturing-induced variation in stiffness of 3D-printed parts [21]. Consequently, the reliability of computational predictions in such systems depends directly on the accurate representation of the printing-orientation-dependent elastic response within the finite element framework.

The finite element method (FEM) is a computational mechanics framework extensively applied across engineering disciplines including structural analysis, materials engineering, and fracture mechanics to solve complex boundary value problems through spatial discretization and numerical approximation of governing equations [22,23,24,25,26,27]. The predictive accuracy of FEM-based simulations is inherently dependent on the fidelity of the constitutive material models implemented, particularly in systems exhibiting anisotropic or heterogeneous behavior [28,29]. Within the field of biomechanics, FEM has been further adopted to evaluate tissue implant interactions, orthopedic constructs, dental restorations, and prosthetic socket–limb mechanics, enabling detailed assessment of internal stress transfer mechanisms that are not directly measurable in vivo [30,31,32,33,34,35,36].

Therefore, this study aims to bridge this gap by conducting a combined experimental and computational investigation. The objectives are threefold: (1) to experimentally determine the tensile properties (Young’s modulus, ultimate tensile strength, elongation at break) of ABS, PLA, and PLA+ printed at four distinct angles (0°, 30°, 60°, 90°); (2) to incorporate the experimentally measured, angle-specific Young’s modulus values as separate equivalent isotropic material cases into a finite element analysis of a patient-specific transtibial prosthetic socket under simplified quasi-static stance loading; and (3) to compare the material and printing angle combinations and identify a balanced configuration based on interfacial pressure, internal stress, and deformation, thereby providing a robust, data-driven framework for the design and manufacturing of high-performance, 3D-printed prosthetic sockets.

## 2. Methodology

The methodology of this study was executed in two integrated phases to evaluate the structural behavior of the prosthetic socket. In the first phase, an experimental investigation was conducted to determine the mechanical properties of three filament materials (ABS, PLA, and PLA+). Tensile test specimens were fabricated at various printing angles (0°, 30°, 60°, and 90°) to capture the printing orientation-dependent mechanical response of 3D-printed polymers. This preliminary phase was crucial for obtaining experimental data, including Young’s modulus and tensile strength, specific to each printing orientation. In the second phase, these experimentally derived properties were used as input parameters for a Finite Element Analysis (FEA) model. A separate simulation was conducted for each material angle combination, and the experimentally determined Young’s modulus was assigned to the socket as an equivalent linear elastic property. By integrating the specific characteristics of each angle and material into the socket’s numerical model, the simulation could comparatively evaluate the stress distribution, displacement, and contact pressure under simplified quasi-static stance loading.

### 2.1. Specimen Preparation and 3D Printing

The tensile specimens (ASTM D638 Type IV [37]) were fabricated using an Anycubic fused filament fabrication (FFF) 3D printer (Anycubic, Shenzhen, China) (Figure 1a). A nozzle with a diameter of 0.2 mm was employed to ensure high dimensional accuracy and consistent filament deposition, with a layer height of 0.20 mm, 100% infill density, and a printing speed of 50 mm/s. The extrusion temperature was set to 210 °C, while the build plate temperature was maintained at 60 °C to promote proper adhesion and reduce warping during printing; these settings were kept uniform across all three materials to isolate the effect of printing orientation without introducing an additional confounding variable. The machine coordinate system was defined with the *Z*-axis representing the build (vertical) direction, while the *X*-axis was aligned with the loading direction of the tensile specimen and the *Y*-axis represented the transverse direction (Figure 1a). To investigate the influence of printing direction on the mechanical behavior, tensile samples were printed at four raster orientations relative to the loading direction, specifically at 0°, 30°, 60°, and 90°. The printing angle (θ) was defined in the XY plane as the angle between the raster direction and the loading direction (*X*-axis), with the different orientations obtained by rotation about the *Z*-axis (build direction), as illustrated in Figure 1a. The geometry and dimensions of the ASTM D638 Type IV tensile specimen are shown in Figure 1b, while representative printed specimens for ABS, PLA, and PLA+ at the four investigated orientations are presented in Figure 1c. All printing parameters were kept constant across the specimens, except for the printing orientation, in order to ensure a reliable comparison of the resulting tensile properties (Figure 1).

### 2.2. Experimental Tensile Testing

Uniaxial tensile tests were performed according to ASTM D638 standards using a ZwickRoell Z400 universal testing machine equipped with a 25 kN load cell (ZwickRoell GmbH & Co. KG, Ulm, Germany). The strain was measured using an extensometer. Stress–strain curves were generated for each specimen type, as shown in Figure 2.

Stress–strain curves obtained from the tensile tests (Figure 2) were used to extract the experimental values for key material properties: Young’s modulus (E), ultimate tensile strength (UTS), and elongation at break (εf). The results demonstrated clear variation in these properties based on the printing angle. Particular focus was placed on Young’s modulus as a primary indicator of stiffness, and its variation across the four printing angles (0°, 30°, 60°, 90°) for the three materials (PLA, PLA+, ABS) is presented in Figure 3, confirming a clear material-specific dependence of Young’s modulus on printing angle. The experimentally determined Young’s modulus for each material orientation combination was subsequently used as the elastic stiffness input in the corresponding finite element simulation.

### 2.3. Finite Element Modeling

#### 2.3.1. Geometric Reconstruction and Materials

The model geometry, comprising the residual limb (tibial bone and surrounding soft tissue), a prosthetic liner, and the custom socket, was adopted from our previously published studies [38,39]. In these works, anatomically representative 3D models were reconstructed from CT-based data using 3D Slicer (Brigham and Women’s Hospital, Harvard Medical School, Boston, MA, USA) to obtain accurate surface geometries (STL format).

In the present study, the previously developed geometrical model was reused without modification of its anatomical features and further processed to design the prosthetic liner and socket using SolidWorks 2017 (Dassault Systèmes, Vélizy-Villacoublay, France) [40]. This approach supports consistency and reproducibility in the comparative biomechanical analysis. The complete reconstruction and design workflow is illustrated in Figure 4.

The experimentally obtained angle-dependent material properties for ABS, PLA, and PLA+ were assigned to the socket model accordingly. For each polymer, four separate finite element simulations were conducted, corresponding to the 0°, 30°, 60°, and 90° printing orientations. In each simulation, the socket was defined as a homogeneous, linear elastic, equivalent isotropic material, and the experimentally determined Young’s modulus of the corresponding material–orientation combination was assigned. The Poisson ratios used for ABS, PLA, and PLA+ were ν = 0.35, 0.36, and 0.35, respectively, and were kept constant across all printing orientations. Therefore, printing orientation was incorporated through the experimentally measured variation in Young’s modulus rather than through a complete orthotropic stiffness matrix or spatially varying local material axes.

It should be clarified that the printing angle (0°, 30°, 60°, 90°) refers to the raster orientation relative to the loading axis of the flat tensile specimens, and does not represent a physically replicated build orientation of the curved socket geometry itself. Given the anatomically complex, doubly curved surface of the socket, a single global printing angle cannot be mapped one-to-one onto such geometry, as the local raster direction would vary continuously across the surface relative to any fixed reference axis, such as the tibial axis. Accordingly, the four angle-dependent Young’s modulus values were treated as four parametric, equivalent-isotropic material cases, used to evaluate the sensitivity of the socket’s structural response to the range of stiffness produced by printing orientation, rather than to simulate four distinct physical printing configurations of the socket. Establishing a direct correspondence between local raster orientation and the tibial axis for an actual patient-specific socket print—and assigning spatially varying orthotropic properties accordingly—is identified as an important direction for future work. Figure 5 illustrates the integration methodology.

The material properties for the biological and liner components in the finite element model were defined as homogeneous, linear elastic, and isotropic. The Young’s modulus and Poisson’s ratio for each structure were assigned based on widely referenced values in prosthetic biomechanics literature: tibial bone (10 GPa, ν = 0.3) [41], soft tissue (0.2 MPa, ν = 0.475) [39], and prosthetic liner (0.38 MPa, ν = 0.39) [40]. These properties remained constant across all simulations to isolate the effect of the socket’s experimentally calibrated, printing-orientation-dependent equivalent elastic response.

#### 2.3.2. Mesh and Boundary Conditions

##### Mesh

To discretize the complex geometry of the transtibial prosthetic socket and the residual limb, a three-dimensional tetrahedral mesh using C3D4 elements (4-node linear tetrahedron) was employed, as illustrated in Figure 6a. This element type was selected for its ability to conform to irregular, patient-specific anatomical contours, including varying socket wall thicknesses and curved surfaces, which are challenging to mesh with structured hexahedral elements, consistent with the majority of published FEA studies on tibial bone and transtibial socket mechanics [20,41,42]. A mesh sensitivity analysis confirmed result convergence, with global stress variations becoming negligible (<2%) when the total element count exceeded 100,000. The final converged mesh consisted of 14,023 elements for the socket, 10,627 elements for the liner, 24,762 elements for the tibial bone, and 58,932 elements for the soft tissue, giving a total of 108,344 elements. The present analysis reports convergence for the C3D4 discretization but does not include a separate C3D10 benchmark or detailed element-quality statistics; therefore, the convergence result supports the comparative global response more strongly than exact local interface stress gradients.

##### Boundary and Loading Conditions

Tie constraints were applied to model the fully bonded interface between bone and soft tissue. Surface-to-surface contact interactions with a friction coefficient of 0.5 [40] were defined between the residual limb and the liner and between the liner and the socket. The distal end of the socket was fully fixed. A static vertical load of 350 N, representing approximately half body weight under a simplified bilateral stance condition, was applied to the apex of the tibial bone [42,43]. This simplified loading condition provides a consistent basis for evaluating interface mechanics and comparative structural performance across material and printing angle configurations, as illustrated in Figure 6b.

## 3. Results and Discussion

### 3.1. Results

Finite element analysis was performed on the transtibial socket model using the experimentally determined Young’s modulus values for ABS, PLA, and PLA+ at each printing orientation. The simulation yielded quantitative data on three key performance indicators: contact pressure at the stump–liner interface, von Mises stress distribution within the socket structure, and overall deformation under load. These results, which demonstrate the influence of both material selection and printing angle on socket mechanics, are presented in the following sections and corresponding figures.

#### 3.1.1. Interface Contact Pressure (CPRESS)

The distribution of contact pressure at the critical stump–liner interface is presented in Figure 7. Across all materials and print orientations, CPRESS was consistently concentrated in three anatomically relevant regions: the lateral tibial head (LTH), the tibial end (TE), and the fibular end (FE). Although minor local intensity variations were observed, the overall spatial pattern remained nearly identical for all configurations.

The similarity in pressure topology indicates that altering the printing angle or material does not substantially modify the primary load transfer pathways at the limb–socket interface.

To quantitatively evaluate these observations, the corresponding peak contact pressure values for all configurations are summarized in Figure 8. The peak CPRESS values remained within a remarkably narrow interval of 0.08911–0.09020 MPa, demonstrating minimal sensitivity to both material type and printing angle.

Although ABS consistently produced the highest peak pressures within this narrow range, followed by PLA+ and PLA, the differences were marginal. The limited variation across print orientations further indicates that modifying build direction affects the internal mechanical response of the socket but does not substantially alter its pressure redistribution performance at the skin interface.

These results indicate that the investigated material and printing orientation combinations had only a limited effect on peak interfacial pressure under the assumptions of the present model.

Table 1 provides a cross-study comparison of peak interface contact pressures (CPRESS), supporting the biomechanical plausibility of the present finite element model. The selected studies were specifically included because they employ comparable finite element approaches involving prosthetic socket and residual limb interface mechanics. The consistency between our predicted pressure range (0.08911–0.09020 MPa) and the spectrum of values reported in these comparable FE studies (0.065–0.125 MPa) supports the biomechanical plausibility of the adopted modeling approach. Importantly, the present results are slightly lower than the maximum contact pressure values reported by Cagle et al. (2018), although these values should not be interpreted as a universal clinical safety threshold [44].

#### 3.1.2. Von Mises Stress Within the Socket Wall

Figure 9 illustrates the von Mises stress distribution throughout the socket structure, serving as an indicator of its internal structural demand under the applied load. A clear mechanical trade-off governed by material compliance is evident from the quantitative results. The stiffest material, PLA, consistently developed the highest internal stresses across all orientations, culminating in a maximum of 2.026 MPa at a 90° printing angle. This represents an approximately 1.1% increase over its stress at 0° of approximately 2.004 MPa.

In stark contrast, the most compliant material, ABS, developed the lowest internal stress levels under the present load case. It achieved the lowest overall stress values, with a minimum of 1.872 MPa at 90°—a value approximately 7.6% lower than the peak stress observed in PLA. This response is consistent with the greater compliance of ABS in the equivalent-isotropic model.

PLA+ exhibited an intermediate stress profile. Its stress values ranged approximately from 1.980 MPa to 1.994 MPa, indicating a relatively stable response across printing angles. This behavior supports its intermediate structural response compared with standard PLA.

#### 3.1.3. Displacement Magnitude (U)

The deformation response of the socket under load is illustrated in Figure 10. The displacement field exhibits a bending-dominated deformation pattern, primarily concentrated in the distal and posterior regions of the socket. While the spatial deformation mode remains consistent across all materials and print orientations, its magnitude varies systematically with material stiffness. This indicates that the experimentally measured, printing orientation-dependent stiffness influences global compliance without fundamentally altering the deformation topology.

The quantitative comparison of the corresponding peak displacement and von Mises stress values is summarized in Figure 11a and Figure 11b, respectively. As shown in Figure 11a, ABS exhibited the highest deformation levels, with displacement generally increasing with printing angle and reaching a maximum of 0.366 mm at 90°. This represents a 32.6% increase relative to the stiffest configuration (PLA at 90°). In contrast, PLA demonstrated the lowest displacement values, decreasing to 0.276 mm at 90°. PLA+ maintained a notably stable deformation response within a narrow range of 0.294 to 0.302 mm (<2.7% variation across angles).

The complementary internal stress response, presented in Figure 11b, reveals the mechanical trade-off governing socket performance. While PLA ensures superior dimensional stability, it exhibits comparatively higher von Mises stresses. Conversely, ABS reduces internal stress at the expense of increased compliance. PLA+ achieves an intermediate stress state while maintaining controlled deformation, quantitatively supporting its balanced printing orientation-dependent response.

To consolidate the quantitative findings presented across Figure 3, Figure 8 and Figure 11 and to provide a transparent, reproducible reference for the experimentally determined material properties and the corresponding peak simulation outputs, Table 2 summarizes the Young’s modulus, ultimate tensile strength, and elongation at break obtained from tensile testing, together with the peak interface contact pressure (CPRESS), peak von Mises stress, and peak displacement magnitude extracted from the finite element simulations, for each material and printing angle combination. These values represent the maximum response recorded across the entire model domain for each configuration and are intended to complement the qualitative color contour comparisons shown in Figure 3, Figure 8 and Figure 11 with discrete, directly comparable numerical data.

### 3.2. Discussion

The integrated experimental–computational approach employed in this study shows that the main effect of the investigated material–orientation cases is a trade-off in the socket structural response rather than a substantial change in peak interface pressure. Interface pressure is governed primarily by the applied load and the limb–liner–socket contact geometry; because the predicted socket deformations remained below 0.4 mm, these conditions changed only slightly among cases. By contrast, internal von Mises stress and displacement respond directly to the assigned socket stiffness, explaining their clearer material- and angle-dependent variation. To place the stress magnitudes in structural context, each peak von Mises stress was compared with the corresponding experimentally measured UTS (Table 2), yielding the UTS-based factors of safety reported in Table 3. These values range from 16.6 for ABS at 90° to 27.2 for PLA at 60°/90°, indicating a large margin relative to the measured UTS under the specific quasi-static load case. Because yield strength was not separately determined in the available tensile dataset, these values should not be interpreted as yield-based safety factors.

As a simple load-scaling sensitivity check, multiplying the present peak stresses by 1.5 would still leave them below the measured UTS for all configurations. However, such scaling does not represent cyclic fatigue, strain-rate effects, impact loading, or the multiaxial load history of gait; therefore, long-term dynamic performance cannot be inferred from the present quasi-static analysis.

The results demonstrate an inverse relationship between internal stress and global deformation. Stiffer sockets (e.g., PLA at high angles) minimize deformation, which may support structural stability, but exhibit higher internal stress levels (Figure 9). Conversely, more compliant sockets (e.g., ABS) reduce internal stress through greater deformation (Figure 10), which may improve load accommodation but increase socket compliance.

This trade-off establishes a clear design framework. For applications demanding greater dimensional stability, a stiffer configuration like PLA at a high printing angle may be suitable. For greater compliance, ABS may be advantageous. However, for a balanced structural response within the present model, PLA+ printed at 0° emerges as a favorable configuration among those investigated. It avoids the higher stress levels of PLA and the greater deformation of ABS, offering a balanced compromise between stress and deformation in the present simulations.

### 3.3. Limitations

The present study has several limitations that should be considered when interpreting the results. First, the finite element analysis was performed under a simplified quasi-static loading condition of 350 N, representing a bilateral stance condition. Therefore, the model does not reproduce the cyclic, multidirectional, and impact-related loading that occurs during gait, and fatigue behavior cannot be inferred from the present simulations. Second, the effect of printing orientation was represented using experimentally measured, orientation-dependent Young’s modulus values within an equivalent isotropic material model. Although this approach enables a controlled comparison of stiffness effects, it does not account for the complete orthotropic behavior of FFF-printed polymers, including orientation-dependent shear properties, Poisson’s ratios, interlayer failure mechanisms, and fracture behavior. In addition, impact toughness, microstructural characteristics, porosity, and interlayer adhesion were not experimentally evaluated in the present study. The use of first-order tetrahedral elements may also limit the resolution of highly localized stress gradients, although mesh convergence was achieved for the global structural response. Finally, the experimental and numerical findings are specific to the investigated materials, printing conditions, and model assumptions, and therefore should not be directly generalized to all FFF systems or clinical loading scenarios. Future studies should incorporate full orthotropic material characterization, dynamic and cyclic gait loading, fatigue and impact testing, microstructural analysis, higher-order finite elements where appropriate, and experimental validation of socket–limb interface pressures using physically manufactured prosthetic sockets.

## 4. Conclusions

This study investigated the effect of printing orientation on the mechanical response of 3D-printed ABS, PLA, and PLA+ materials and examined how the experimentally determined orientation-dependent Young’s modulus values influence the structural behavior of a patient-specific transtibial prosthetic socket through finite element analysis. The combined experimental and computational results provide a direct comparison of material stiffness, internal stress, deformation, and interface contact pressure across the investigated material and printing angle combinations. The main findings of the study are summarized as follows:

1. Printing orientation produced clear material-specific variations in the experimentally determined mechanical properties of ABS, PLA, and PLA+, demonstrating the importance of considering orientation-dependent stiffness in 3D-printed prosthetic socket design.
2. PLA exhibited the lowest deformation, reaching approximately 0.276 mm, but developed the highest von Mises stress of up to 2.026 MPa. In contrast, ABS showed the lowest internal stress, approximately 1.872 MPa, together with the highest deformation of approximately 0.366 mm.
3. PLA+ exhibited a comparatively balanced and stable structural response across the investigated orientations. In particular, PLA+ printed at 0° showed approximately 0.297 mm displacement and 1.989 MPa von Mises stress, representing a favorable compromise among the evaluated configurations.
4. Peak interface contact pressure remained within a narrow range of 0.08911 to 0.09020 MPa for all material and printing angle combinations. This indicates that the investigated orientation-dependent elastic properties had only a limited influence on peak interfacial pressure under the assumptions of the present finite element model.

## Figures and Tables

**Figure 1:**
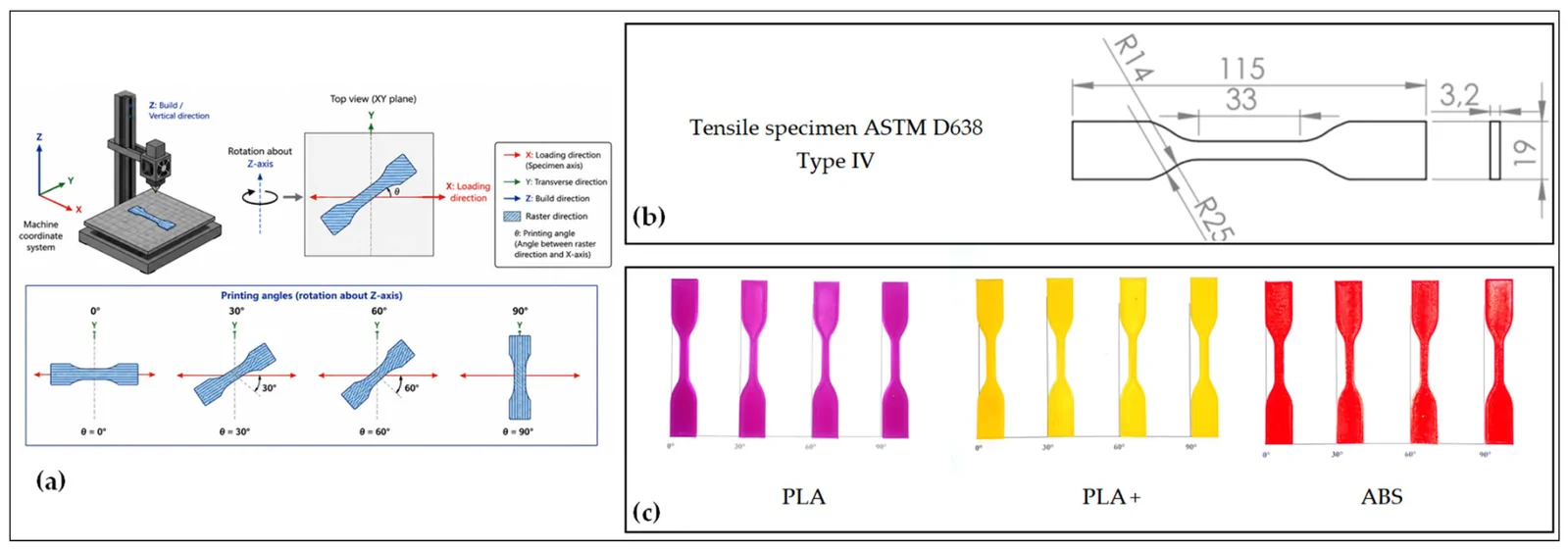
(**a**) Machine coordinate system and four printing orientations (0°, 30°, 60°, and 90°), showing the raster and loading directions and rotation about the *Z*-axis; (**b**) geometry and dimensions of the ASTM D638 Type IV specimen; and (**c**) representative 3D-printed specimens of ABS, PLA, and PLA+ at the four investigated orientations.

**Figure 2:**
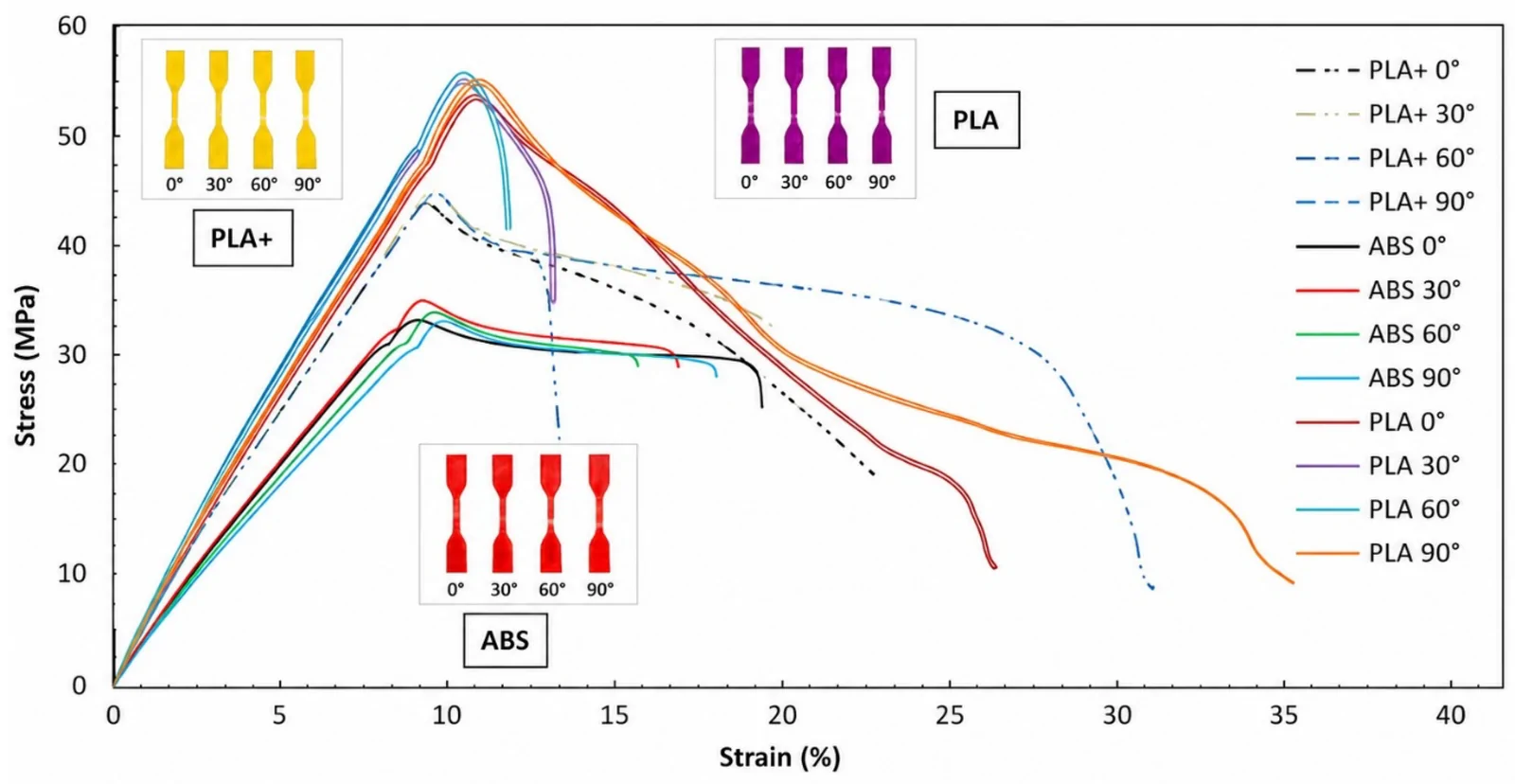
Stress–strain curves of 3D-printed tensile specimens (PLA, PLA+, and ABS) at different printing orientations (0°, 30°, 60°, and 90°).

**Figure 3:**
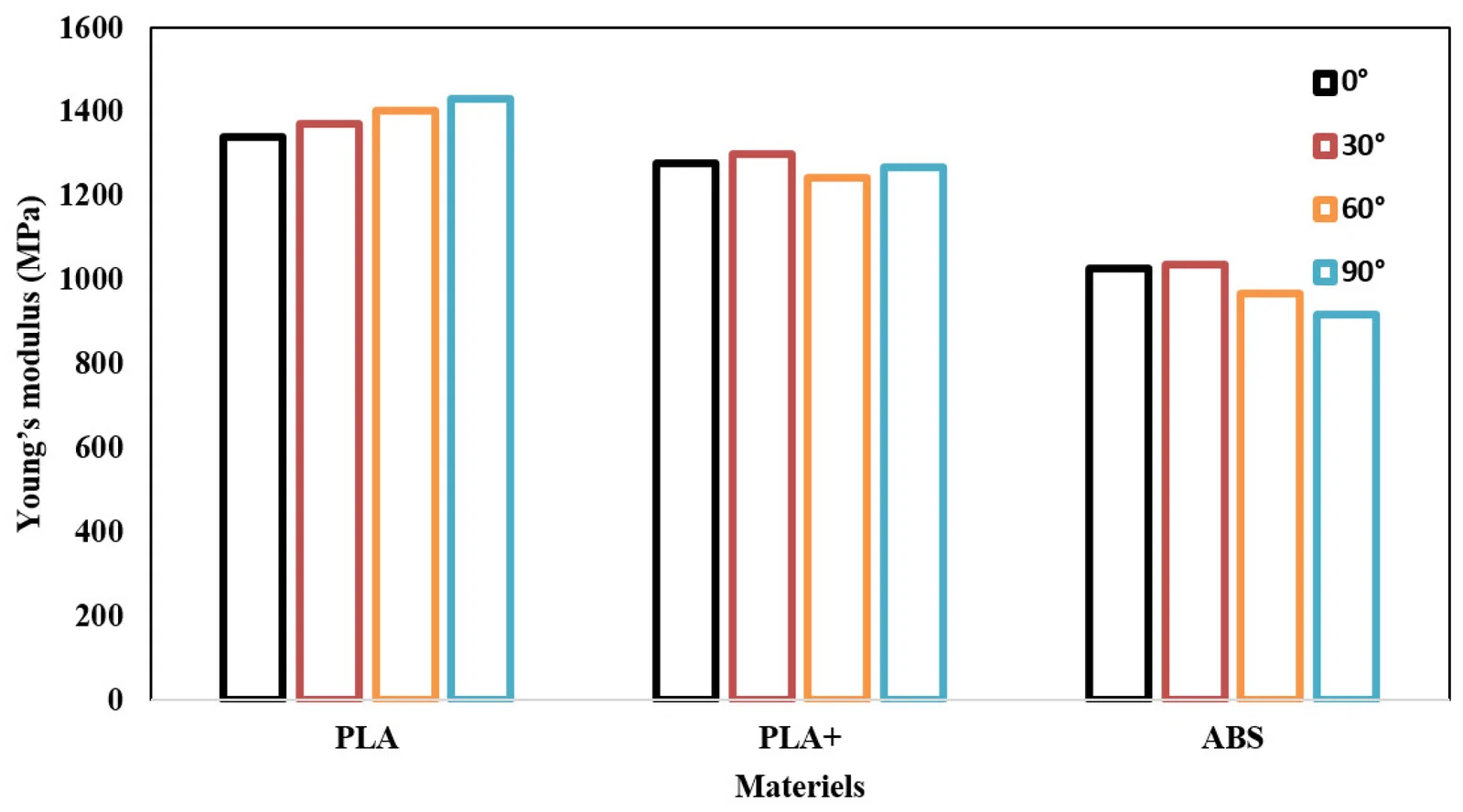
Variation in Young’s modulus for PLA, PLA+, and ABS materials at different 3D printing orientations (0°, 30°, 60°, and 90°).

**Figure 4:**
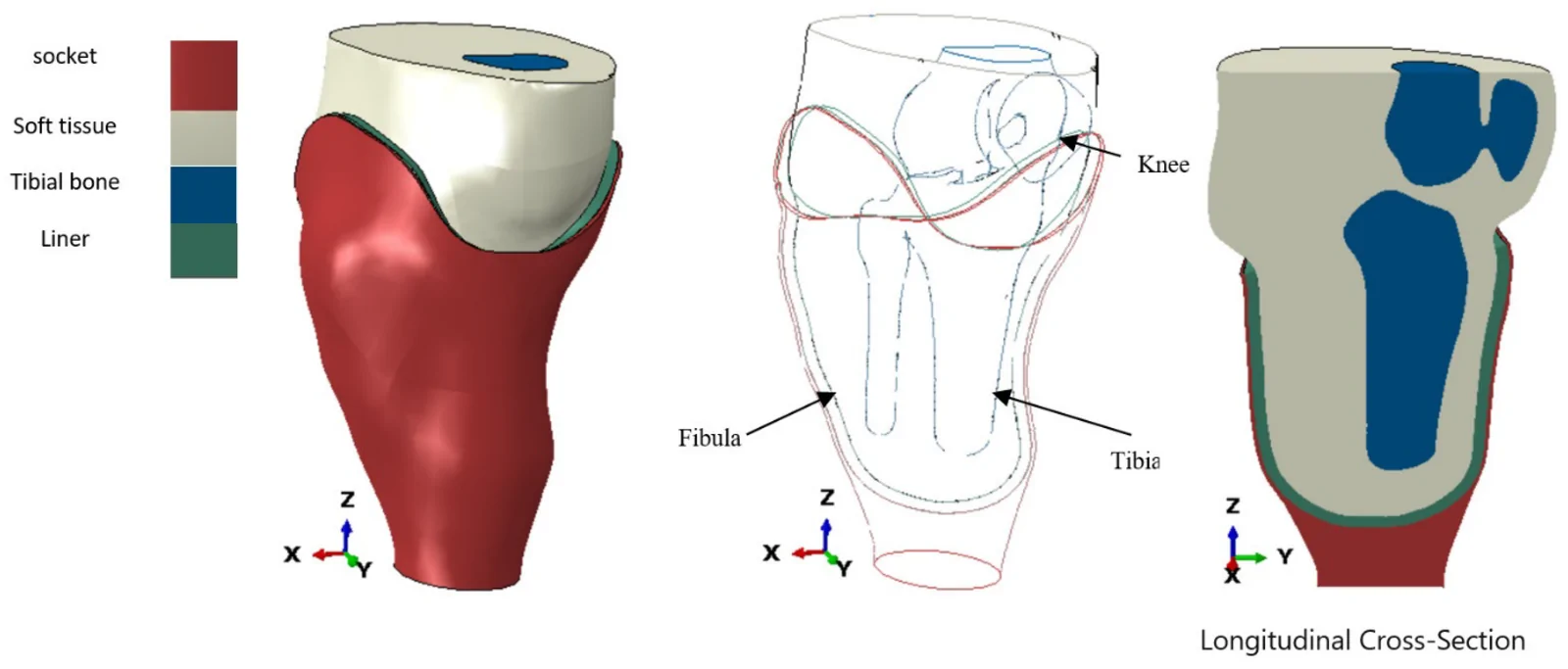
Detailed geometry of the finite element model.

**Figure 5:**
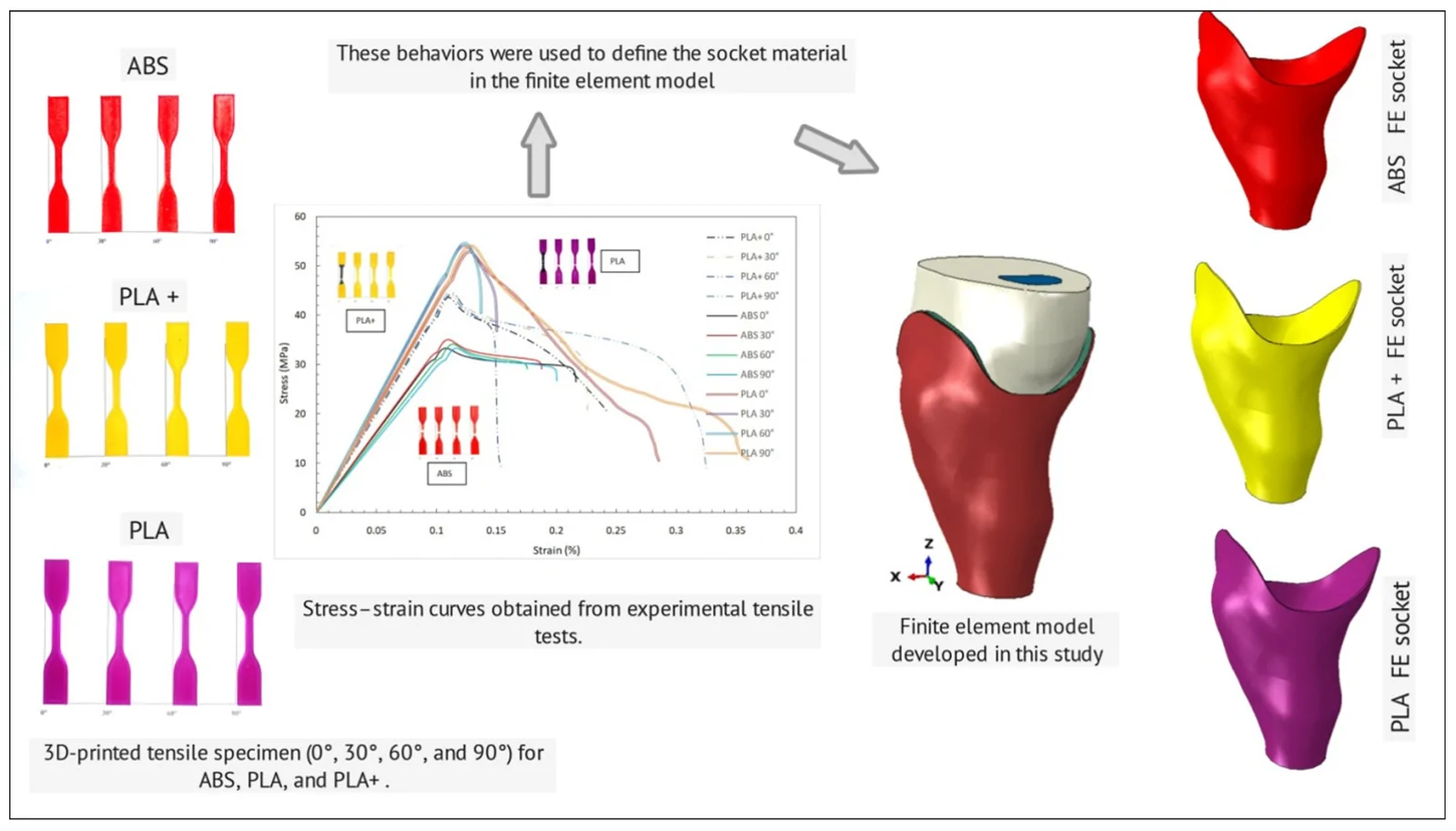
Methodological framework for integrating experimentally determined, printing-orientation-dependent Young’s modulus values into the FEA socket model.

**Figure 6:**
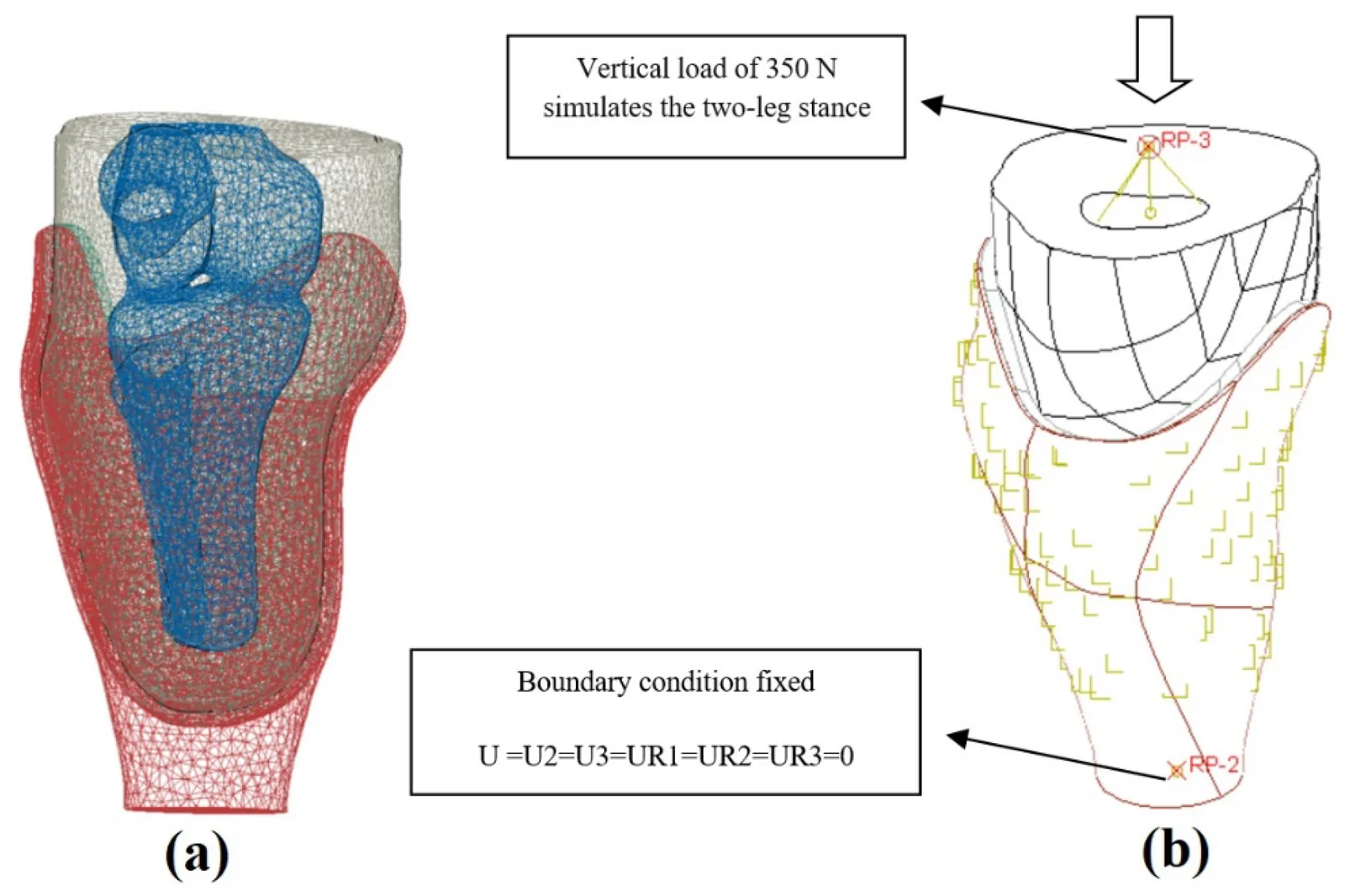
(**a**) Three-dimensional finite element mesh models and (**b**) applied boundary conditions and loading configuration.

**Figure 7:**
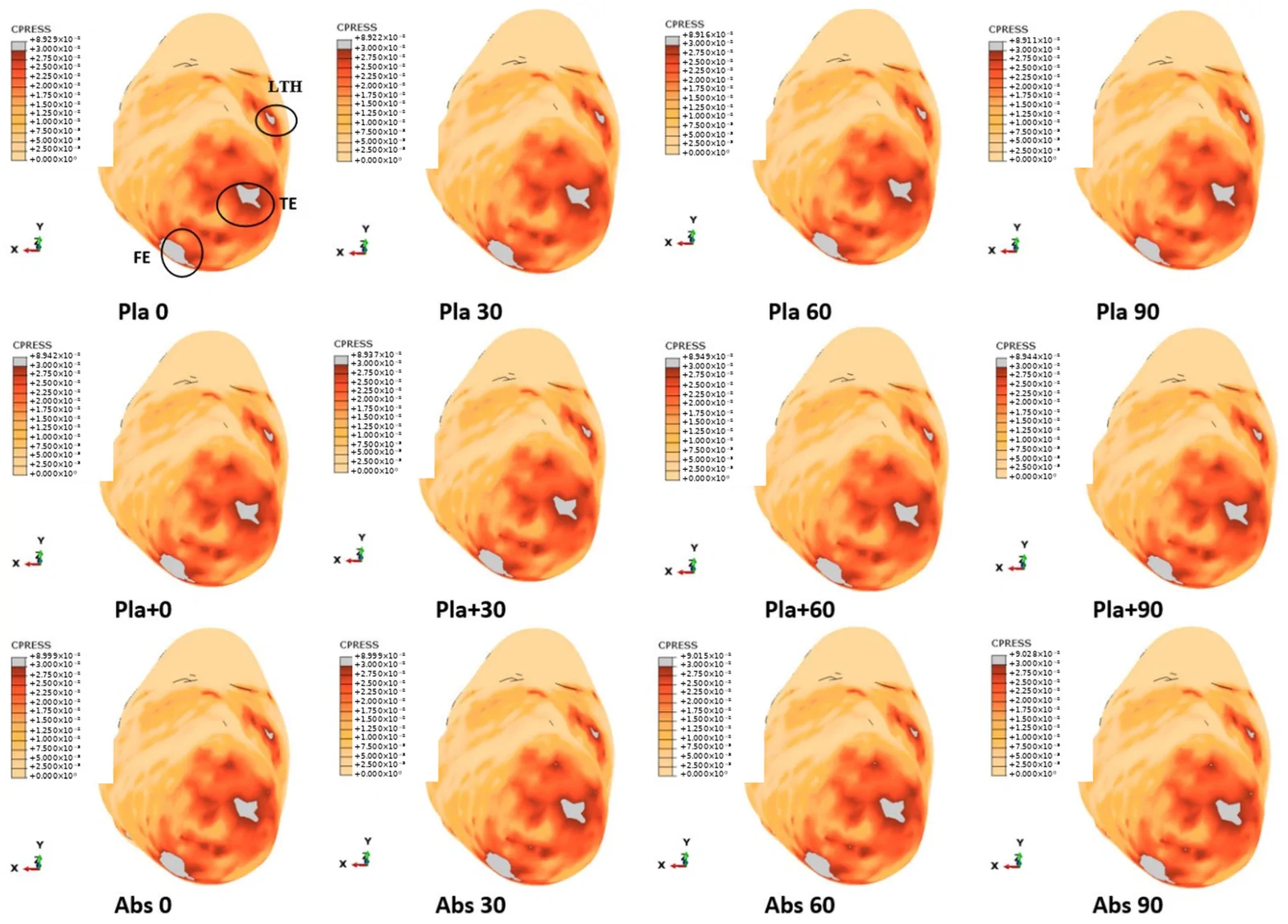
Contact pressure (CPRESS) distribution at the stump–liner interface.

**Figure 8:**
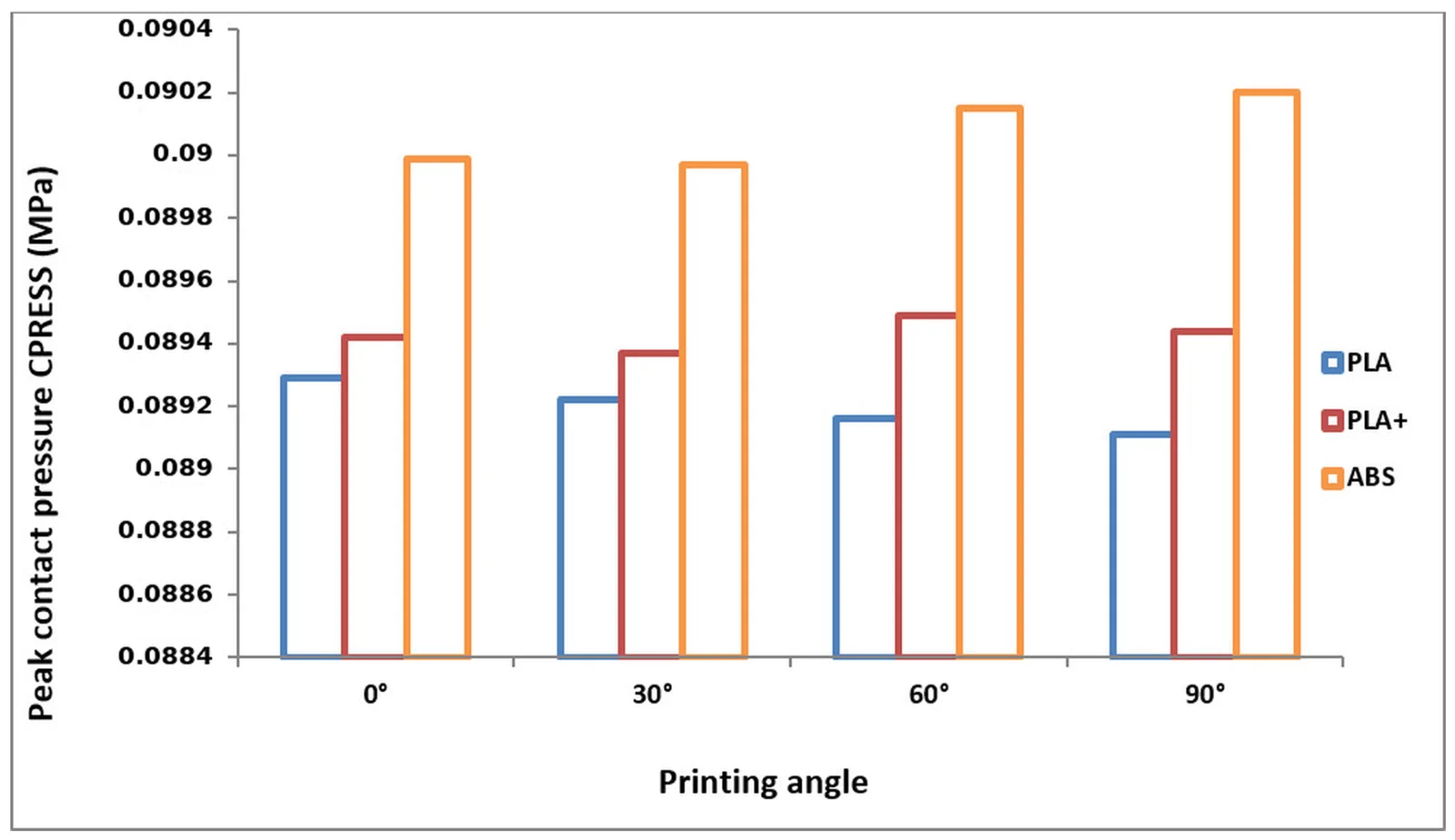
Peak contact pressure (CPRESS) at the stump–liner interface across different materials and printing orientations.

**Figure 9:**
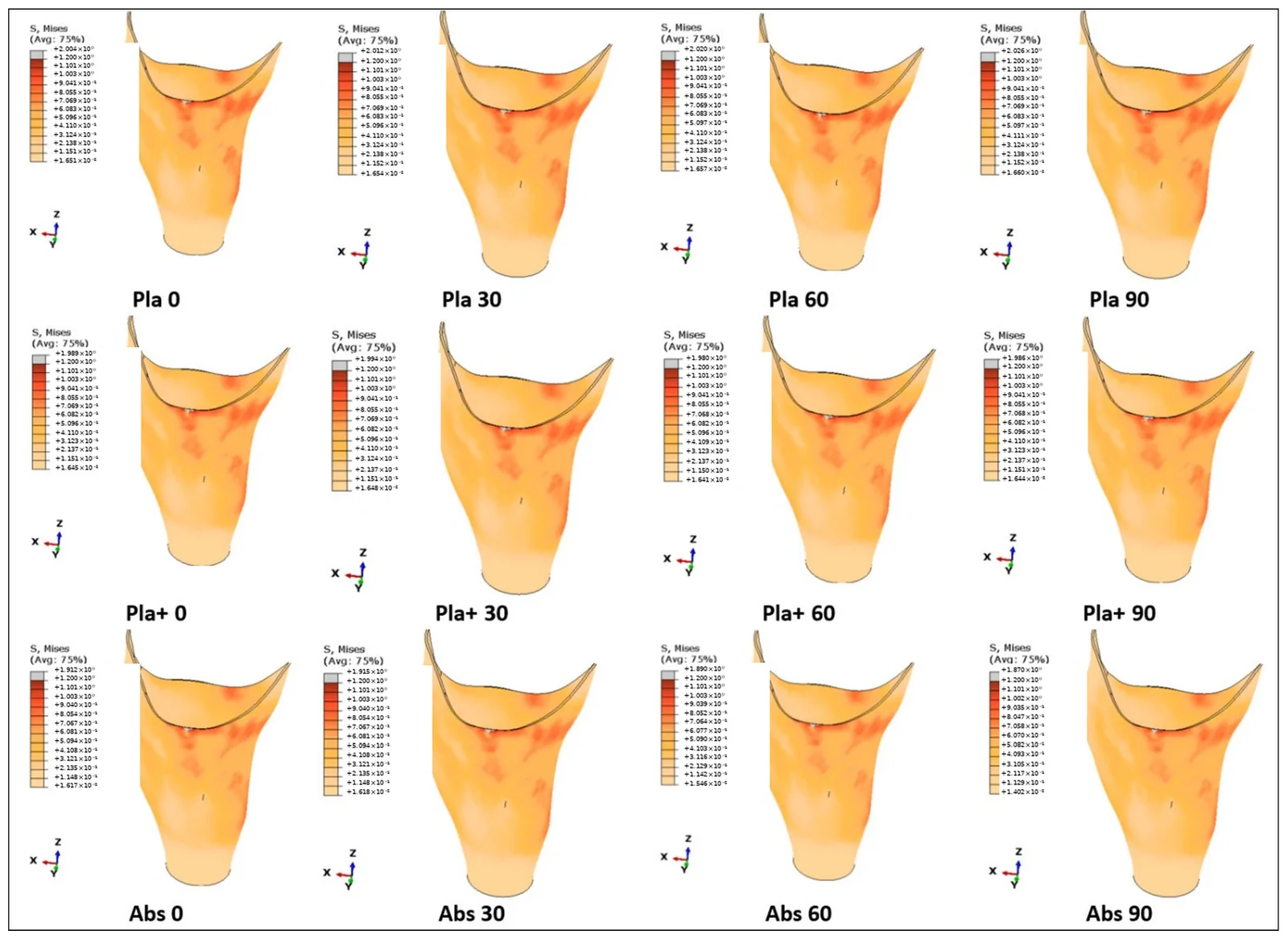
Von Mises stress distribution within the prosthetic socket for all material and printing angle combinations.

**Figure 10:**
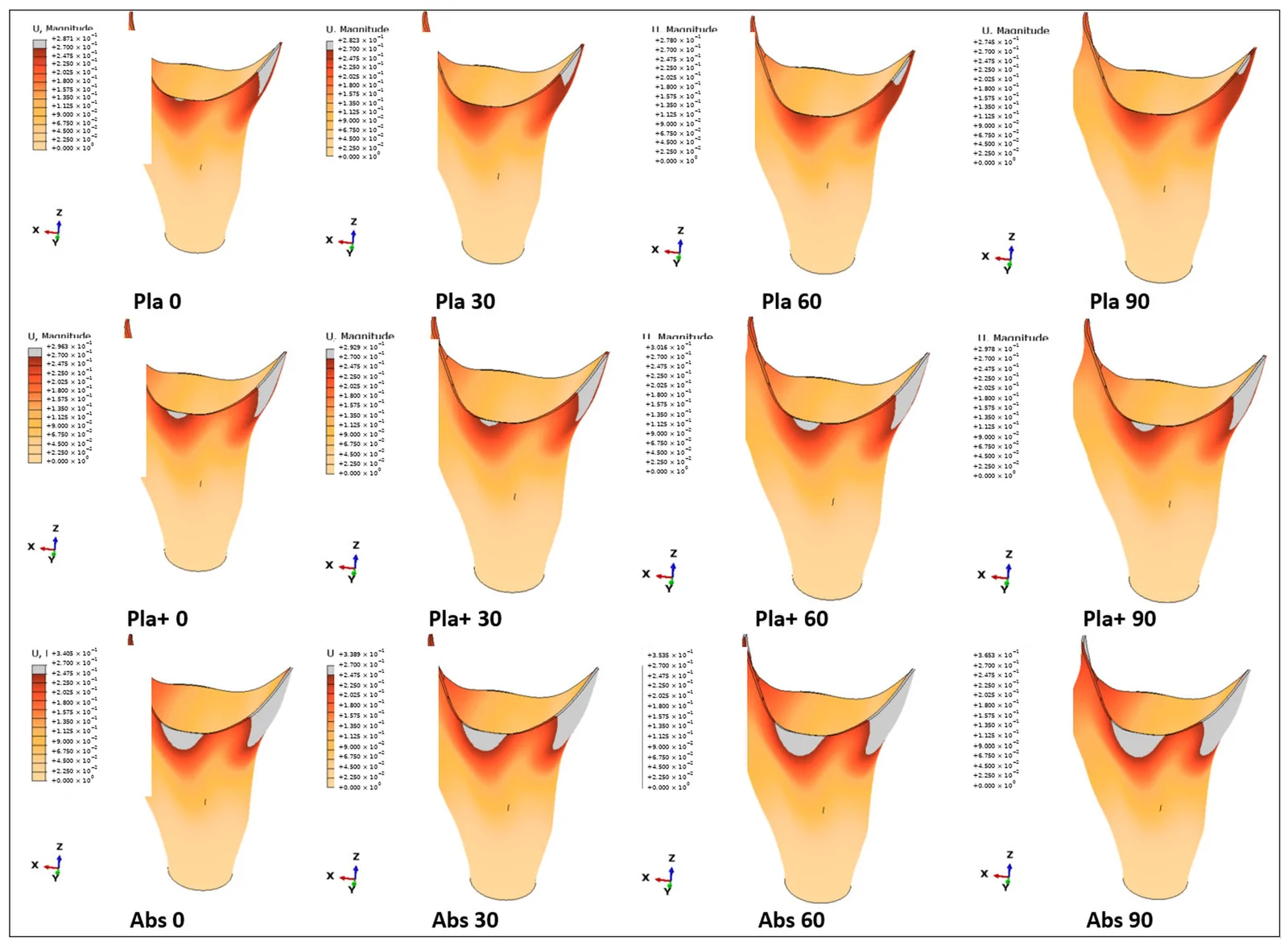
Displacement magnitude (U) distribution within the prosthetic socket for all material and printing angle combinations.

**Figure 11:**
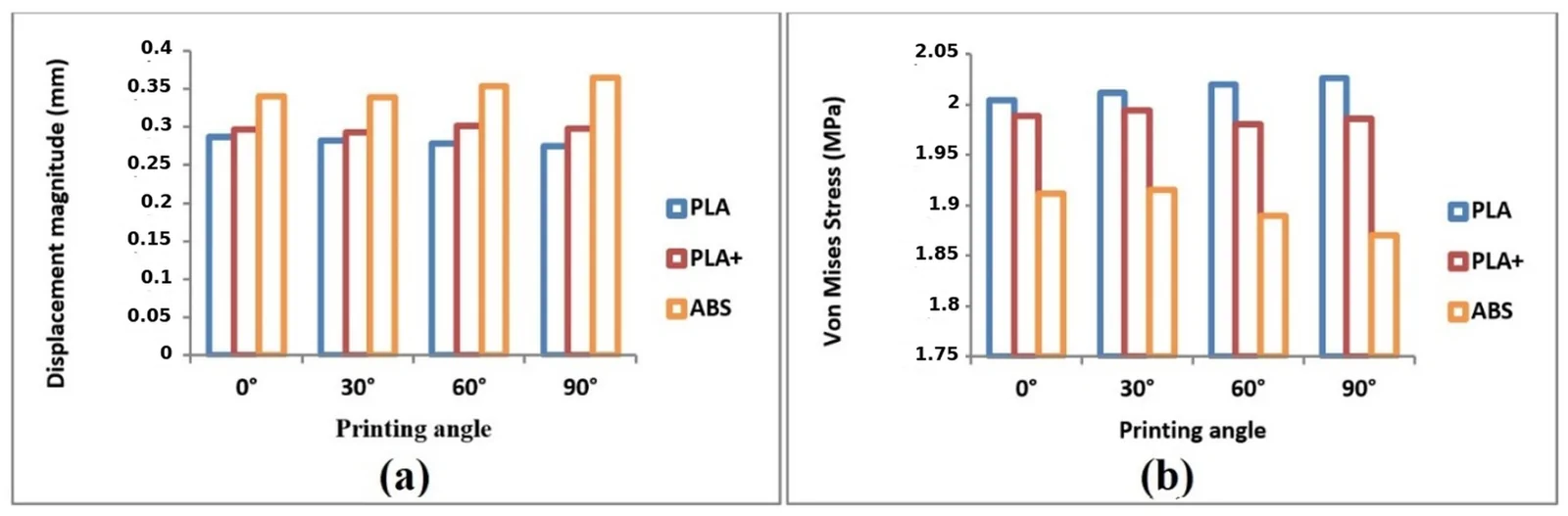
Structural response of the prosthetic socket: (**a**) peak displacement magnitude (mm) and (**b**) von Mises stress distribution (MPa).

**Table 1:** Cross-study comparison of peak CPRESS values in transtibial socket FE models.

Reference Source	Method/Context	Reported Peak CPRESS (MPa)	Current Study (MPa)	Deviation %
Sampaio et al. (2025) [20]	FE socket limb interface (patient specific)	0.125	0.08911–0.09020	▼ 28.27%
Portnoy et al. (2009) [43]	FE pressure mapping with clinical residual limb data	0.065	0.08911–0.09020	▲ 37.94%
Cagle et al. (2018) [44]	TSB socket FE model (patient geometry)	0.095–0.098	0.08911–0.09020	▼ ~8%
Current study	FE with experimentally calibrated Young’s modulus values	—	0.08911–0.09020	—

Note: ▼ indicates lower and ▲ indicates higher values relative to the reference study.

**Table 2:** Summary of experimentally determined tensile properties (Young’s modulus, ultimate tensile strength, elongation at break) and peak finite element simulation outputs (interface contact pressure, von Mises stress, displacement magnitude) for PLA, PLA+, and ABS at printing angles of 0°, 30°, 60°, and 90°.

Material	Angle	E (MPa)	UTS (MPa)	Elongation (%)	Peak CPRESS at Liner–Residual Limb Interface (MPa)	Peak von Mises Stress in Socket (MPa)	Peak Displacement in Socket (mm)
PLA	0°	1338.11	53	28	0.0893	2.004	0.2871
PLA	30°	1372.16	53	20	0.0892	2.012	0.2823
PLA	60°	1403.41	55	15	0.0892	2.020	0.2780
PLA	90°	1429.85	55	36	0.0891	2.026	0.2745
PLA+	0°	1276.23	44	22.5	0.0894	1.989	0.2963
PLA+	30°	1298.54	46	22.5	0.0894	1.994	0.2929
PLA+	60°	1242.42	46	15.5	0.0895	1.980	0.3016
PLA+	90°	1266.21	44	32.5	0.0894	1.986	0.2978
ABS	0°	1028.19	33	21	0.0900	1.912	0.3405
ABS	30°	1035.70	35	19	0.0900	1.915	0.3389
ABS	60°	967.46	32	17.5	0.0902	1.890	0.3535
ABS	90°	917.52	31	20	0.0903	1.870	0.3653

**Table 3:** Factor of safety (UTS/peak von Mises stress) for each material and printing angle combination.

Material	Angle	UTS (MPa)	Peak von Mises (MPa)	Factor of Safety
PLA	0°	53	2.004	26.4
PLA	30°	53	2.012	26.3
PLA	60°	55	2.020	27.2
PLA	90°	55	2.026	27.2
PLA+	0°	44	1.989	22.1
PLA+	30°	46	1.994	23.1
PLA+	60°	46	1.980	23.2
PLA+	90°	44	1.986	22.2
ABS	0°	33	1.912	17.3
ABS	30°	35	1.915	18.3
ABS	60°	32	1.890	16.9
ABS	90°	31	1.870	16.6

## Data Availability

The data that support the findings of this study are available on request from the authors.
